# Supervised, Heavy Resistance Training Is Tolerated and Potentially Beneficial in Women with Knee Pain and Knee Joint Hypermobility: A Case Series

**DOI:** 10.1155/2022/8367134

**Published:** 2022-12-30

**Authors:** Peter Henriksen, Tina Junge, Jens Bojsen-Møller, Birgit Juul-Kristensen, Jonas Bloch Thorlund

**Affiliations:** ^1^Health Sciences Research Centre, UCL University College, Odense, Denmark; ^2^Department of Sports Science and Clinical Biomechanics, University of Southern Denmark, Odense, Denmark; ^3^Research Unit for General Practice, Department of Public Health, University of Southern Denmark, Odense, Denmark

## Abstract

**Introduction:**

Adults with generalised joint hypermobility including knee joint hypermobility (GJHk) report more knee joint symptoms when compared to adults without GJHk. There is no consensus on best practice for symptom management. For instance, controversy exists regarding the appropriateness and safety of heavy resistance training as an intervention for this specific group. This case series aims to describe a supervised, progressive heavy resistance training program in adults with GJHk and knee pain, the tolerability of the intervention, and the outcomes of knee pain, knee-related quality of life, muscle strength, proprioception, and patellar tendon stiffness through a 12-week period.

**Materials and Methods:**

Adults with GJHk and knee pain were recruited to perform supervised, progressive heavy resistance training twice a week for 12 weeks. The main outcome was the tolerability of the intervention. Secondary outcomes were knee pain during a self-nominated activity (VAS_NA_); Knee injury and Osteoarthritis Outcome Score (KOOS); Tampa Scale of Kinesiophobia (TSK); maximal quadriceps voluntary isometric contraction and rate of torque development; 5 repetition maximum strength in five different leg exercises; single leg hop for distance; knee proprioception and patellar tendon stiffness.

**Results:**

In total, 16 women (24.2 years, SD 2.5) completed at least 21/24 training sessions. No major adverse events were observed. On average, VAS_NA_ decreased by 32.5 mm (95% CI 21.4–43.6), in addition to improvements in KOOS and TSK scores. These improvements were supported by an increase in all measures of lower extremity muscle strength, knee proprioception, and patellar tendon stiffness.

**Conclusion:**

Supervised heavy resistance training seems to be well tolerated and potentially beneficial in young women with GJHk and knee pain.

## 1. Background

Generalised joint hypermobility (GJH) is described as the ability to move several synovial joints beyond the expected range of motion [1]. A recent population-based Danish survey among adults found a prevalence of 13% for self-reported GJH including knee joint hypermobility (GJHk), mainly in women (80%) [2]. Furthermore, adults with GJHk had twofold increased odds of reporting knee joint symptoms (pain, ache, and discomfort) compared to adults without GJHk [2].

Little is known about the etiology of musculoskeletal symptoms in individuals with GJHk. When examining factors related to active knee joint stability in individuals with different hypermobility spectrum disorders, results are inconclusive on force characteristics [3–7] and tendon stiffness [3, 8–11], this may partly be explained by differences in methods and populations. However, impaired knee proprioception seems to be a consistent finding [12–14]. Also, altered knee joint neuromuscular control, i.e., different recruitment pattern of the lower extremity muscles, assessed by EMG activity, is reported in children with nonsymptomatic GJHk during jumping [15] and in women with asymptomatic GJHk when stair climbing [16].

Currently, there is no consensus on the best management of musculoskeletal symptoms in individuals with symptomatic knee joint hypermobility [17, 18]. Usual care typically aims to address joint instability and typically consists of different combinations of proprioceptive training, plyometrics, and light resistance training, showing positive effects on knee pain [12, 14, 19], muscle strength [19], and knee proprioception [12, 14, 19]. Increasing muscle strength is recommended [5, 17], and individuals with hypermobility seem to strengthen at similar rates as controls after 16 weeks of resistance training [6]. However, the relatively low loads typically applied might be suboptimal for targeting all elements in active joint stability for individuals with GJHk. A specific method for targeting and improving active joint stability and decreasing knee pain could be heavy resistance training, as it has been shown to increase muscle cross-sectional area [20], neural drive [21], and tendon stiffness [22, 23] in both healthy and various patient populations but is not previously applied to individuals with GJHk.

The primary objectives of this case series were to provide a detailed description of a 12-week supervised, progressive heavy resistance training program in adults with generalised joint hypermobility including knee joint hypermobility (GJHk) and knee pain, aimed at improving active knee joint stability, as well as evaluating the tolerability (and safety) of the intervention and potential effects on participant reported outcomes (knee pain, knee-related quality of life, and kinesiophobia) and objective outcomes (lower extremity muscle strength, knee proprioception, and patellar tendon stiffness).

## 2. Materials and Methods

### 2.1. Design and Participants

The current study is a case series. The intervention is reported according to the TIDieR guidelines [24]. The heavy strength training program is described according to the CERT guidelines [25]. The study was approved by the Regional Scientific Ethics Committee for Southern Denmark (jnr. S-20170052 HJD/csf) and reported to the Danish Data Protection Agency. Written informed consent was obtained from all study participants.

Participants were recruited via advertising on social media and posters at two higher educational institutions. Inclusion criteria were adults aged 18–50 years with GJHk, persistent knee pain, and self-reported knee pain of a minimum of 30 mm on a 0–100 mm visual analogue scale (VAS) in their most hypermobile knee joint during an activity prenominated as being the most aggravating for the present knee pain (i.e., VAS nominated activity–VAS_NA_) [26].

GJHk was categorised according to the Beighton tests (BT) for general joint hypermobility. The BT consists of nine tests; four bilateral tests of hyperextension of the first and fifth fingers, elbows, and knees; and one unilateral test of forward bending, with a recommended cut point of five or more positive tests out of nine [1]. To ensure knee joint hypermobility, at least one BT for knee joint hypermobility had to be positive, i.e., more than 10 degrees of knee joint hyperextension when standing; in our case also confirmed in a supine position with a goniometer.

Exclusion criteria were known rheumatic or neurological diseases, diagnosed patellar tendinopathy, a body height exceeding 190 cm (for technical reasons related to tendon stiffness assessments), pregnancy, childbirth within the past year, knee surgery within the past year, participation in regular systematic resistance training within the past six months or inability to speak and understand Danish.

### 2.2. Procedures

All participants were assessed for study eligibility during a clinical examination, including a full knee examination by an experienced physiotherapist. Baseline testing was performed within one week of inclusion. First, participants filled out questionnaires, followed by anthropometric measurements and proprioception testing. A standardised warm-up protocol was then completed followed by strength tests and patellar tendon stiffness assessment, a typical testing session lasting 1.5 hours. After completing 12 weeks of supervised, progressive heavy resistance training, all outcome measurements were obtained in the same order, at the same time of the day (±1.5 hours), and by the same tester, to minimize the potential impact of the test order, daily variations, and tester [27]. All participants were instructed not to use pain medication on the days of baseline and follow-up testing.

### 2.3. Intervention

Within six days after baseline testing, participants commenced the supervised, progressive heavy resistance training program (Appendix A) designed according to the American College of Sports Medicine guidelines [28]. The program consisted of five exercises: leg press, resisted sitting calf raise, leg extension, leg curl, and forward lunge. The individual initial load was determined based on a five-repetition maximum (5RM) strength test. Throughout the intervention period, workloads were constantly regulated to meet the RM goals for the given week, and every set was performed to failure, except during the familiarization and tapering weeks. Training frequency was twice a week for 12 weeks with at least 48 hours between sessions. All training sessions were supervised by trained final-year physiotherapy students or the first author of the current study. The training was individual, but participants typically attended the university weight room in small groups and were allowed to encourage each other to increase overall motivation. Completing 21/24 target sessions was considered good adherence. Participants reported their immediate knee pain before and after each training session, using the numeric rating scale (NRS) ranging from zero (no pain) to 10 (worst pain imaginable) [29] making it possible to monitor acute pain flares induced by each training session. Furthermore, knee pain NRS lower than or equal to five was considered acceptable, whereas knee pain NRS higher than five, or contraindications like swelling of the knee, resulted in an adjustment of exercise intensity in the subsequent session, and referral to a clinical examination by the physiotherapist.

### 2.4. Outcomes

#### 2.4.1. Tolerability of the Intervention

Dropout rate (including reasons), number of accomplished training sessions, mean pain flares induced by each training session, adverse events, and possible adjustments to the training program are reported.

#### 2.4.2. Knee Pain

Besides evaluating tolerability, the primary exploratory outcome was self-reported knee pain during an activity, nominated by the participant to be the most aggravating for their present knee pain (VAS_NA_) [26] in the participants' most hypermobile knee. The participants also rated their average knee pain during the last week (VAS_LW_) [26]. VAS scores were obtained by 100 mm VAS, ranging from zero (no pain at all) to 100 (worst pain imaginable) [26].

#### 2.4.3. Knee-Related Quality of Life

Self-reportedknee-related quality of life was assessed by the knee injury and osteoarthritis outcome score (KOOS), a questionnaire consisting of five subscales: pain, other symptoms, function in daily living (ADL), function in sport and recreation (Sport/Rec), and knee-related quality of life (QOL) [30].

#### 2.4.4. Fear of Movement

Fear of movement was assessed by the Tampa Scale of Kinesiophobia (TSK) [31].

#### 2.4.5. Proprioception

A sitting active-active knee joint position reproduction test was performed as a measure of proprioception, expressed as mean absolute angle error (AAE) [12, 32]. Participants were seated on an elevated couch, and an electric goniometer (Biometrics Ltd, NP11, UK) was attached to the lateral side of the knee on the test leg (i.e., the leg with the most hypermobile knee joint). The participants were blindfolded and were asked to actively extend their knees while guided to a predetermined angle position and to hold that position for five seconds. After returning the leg to a passively hanging position, the participants were asked to actively replicate the joint angle. The test was performed twice at 30°, 50°, and 70° degrees, respectively (0° = fully extended, randomized order). Finally, the mean AAE of the six attempts was noted.

#### 2.4.6. Single-Leg Hop for Distance (SLHD)

A modified SLHD test [15, 33] was performed, allowing arm swing assistance to increase both participant confidence and safety. The participants were instructed to hop forward as far as possible and land steadily and stand still for at least three seconds. Following two submaximal practice hops, three hops (separated by 1 min) were performed. Additional hops were allowed until no further advancement in hop length was observed [15]. The longest hop measured in cm from the toe in the starting position to the heel in the landing position was used for analysis.

#### 2.4.7. Knee Extensor Isometric Maximal Voluntary Contraction (MVC) and Rate of Torque Development (RTD)

MVC and RTD were assessed using a customized setup. Participants were positioned in a rigid custom-built chair with the hip and knee joints positioned in 90° flexion, firmly strapped at the hip and the distal part of the thigh. A strain gauge was mounted perpendicular to the lower leg, 3.5 cm proximal to the lateral malleolus via a steal rod and a cuff. The external moment arm was measured as the distance from the center of the cuff to the lateral aspect of the knee joint line. Following several submaximal contractions, isometric knee extensor MVC was performed with visual feedback of the force signal (3 attempts with a 3 min break between trials). Participants were asked to contract as fast and powerfully as possible during maximal contractions [34].

Maximal MVC and RTD were obtained from the trial with the highest peak moment and RTD during the initial 200 ms, respectively (details in Appendix B); the onset of muscle contraction was defined according to previous studies [34].

Dynamic strength (5RM) was established 2–4 days after baseline testing by a standardised protocol [35]. All settings on the training devices (Technogym, Selection Line) were noted and replicated for follow-up testing. The 5RM test was conducted for four out of the five exercises included in the training program (leg press, resisted sitting calf raise, leg extension, and leg curl). Forward lunges were excluded from the 5RM testing as some participants were unable to perform this exercise safely. The 5RM follow-up testing was performed two or three days after the final training session.

#### 2.4.8. Patellar Tendon Stiffness

Stiffness of the patellar tendon was assessed based on corresponding values of tendon force and tendon deformation (measured by use of B-mode ultrasonography), as previously described [36–38]. Participants kept their seated position in the dynamometer, and a 15 MHz linear ultrasound (US) transducer with a 50 mm field of view (Logiq S7, GE Healthcare, Milwaukee, USA) was positioned anteriorly on the knee, ensuring that the distal part of the patella, the patellar tendon, and the proximal part of the tibia were visible within the US field, hereby enabling recording of tendon length changes during linear ramped (7–10 sec) knee extension MVC [36]. US recordings were sampled at 38 Hz, and a custom-built trigger device ensured the synchronisation of US and force data recordings. To ensure a consistent strain rate, a reference image of the target (force-time) slope was presented, and real-time visual feedback of the force signal was provided [37]. The internal moment arm was estimated from the individually measured length of the femur [36, 39] to calculate the tendon force. The tendon elongation was tracked frame by frame twice on each US recording using specific software (Tracker 5.0.5., Open Source Physics), and an average of the two trackings was used for further analysis. A second-order polynomial fit was applied to the force-deformation plot, and tendon stiffness (N/mm) was then determined as the slope of the final 10% of the plot [36, 38].

#### 2.4.9. Sample Size Calculation

This study was intended as preparatory work for a future randomized controlled trial; therefore, we estimated the needed sample size for the intended primary outcome of the trial, VAS_NA_. The minimal clinically important improvement in knee pain is suggested to be between 11 and 37 mm on the VASNA [40]. A sample of 16 participants was needed to detect a within-group change of 15 mm on the VAS_NA_ scale, assuming a SD of 20 mm in the VAS_NA_ [40], a power of 80%, and a significance level of 0.05. Including a 20% dropout rate, at least 20 participants were preferable.

#### 2.4.10. Statistics

Descriptive characteristics are reported with mean and SD and relative numbers when appropriate. Paired *t*-tests were used to determine differences between baseline and follow-up for all observed exploratory outcomes to provide estimates around the observed changes. All statistical analyses were performed in STATA/IC 16.0 (StataCorp. 2019. Stata Statistical Software: release 16. College Station, TX: StataCorp LLC).

## 3. Results

**Figure 1:**
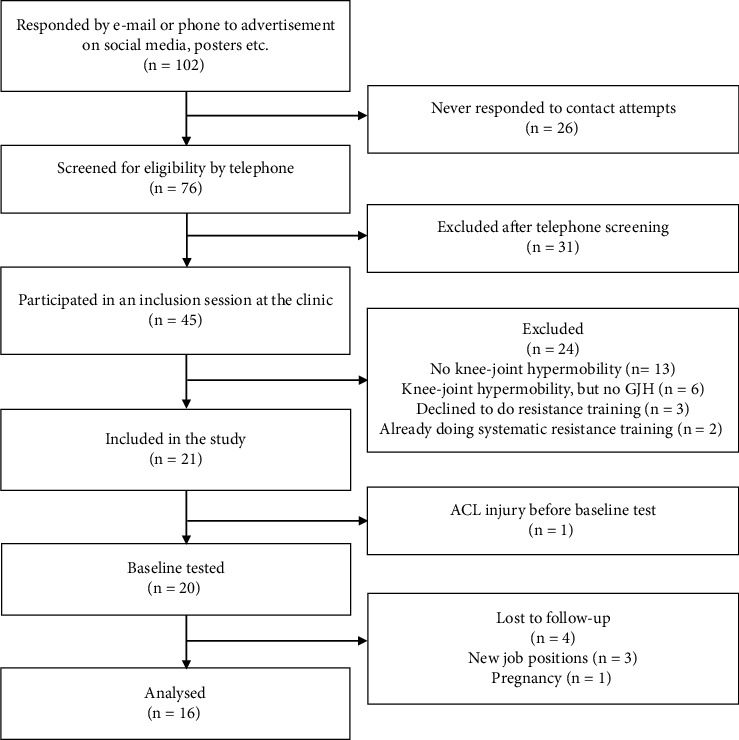
Study flowchart.

Of the 21 participants initially recruited, 16 participants completed the intervention with good compliance (i.e., completed at least 21/24 sessions). One participant dropped out prior to baseline testing due to an anterior cruciate ligament injury (not related to the current study), and four participants dropped out during the study due to changed job situations and in one case pregnancy. This corresponds to a dropout rate of 19% (Figure 1).

**Figure 2:**
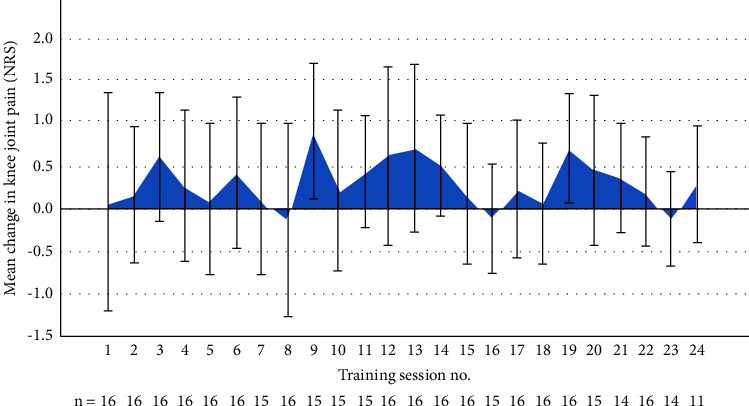
Change in acute knee joint pain from before and straight after each of the 24 training sessions. NRS ranging from 0 to 10. Error bars are 95% CI, *n* = number of available data at each session.

No major adverse events were reported. The mean acute pain flares induced by each training session are presented in Figure 2. In total, 96% of the total possible baseline and follow-up NRS ratings were available.

All participants completing the study were women, and individual and mean descriptive data are presented in Table 1. Mean age was 24 years, mean Beighton score was 6.4 points (SD 1.3), and the average knee joint hyperextension was 11.7 degrees (SD 1.3) for the most hypermobile knee joints. At baseline, 20% of the baseline-tested participants had unilateral knee joint hypermobility, and the remaining 80% had bilateral knee joint hypermobility. The most common activities, nominated as being most aggravating for the participants' present knee pain, were prolonged standing, followed by stair climbing and running/walking (Table 1).

All 16 participants reported decreased knee VAS_NA_ at follow-up (Table 2), and on average, a decrease of 32.5 mm (95% CI 21.4–43.6) was observed (Table 3). Also, on average, improvements were observed for VAS_LW_, several of the KOOS subscales, and TSK, although there was individual variance (Table 3). All participants increased their quadriceps MVC (Table 4), and on average, all other strength measures, with the largest mean improvements (39–82%) in the 5RM tests (Table 5). Similarly, tests for knee proprioception improved in all 16 participants (Table 4), indicated by a mean relative reduction of AAE of 55%, corresponding to 2.2 degrees (95% CI 1.6–2.9) (Table 5). Furthermore, patellar tendon stiffness increased in 11 out of 16 participants (Table 4), causing a mean increase of 11% (280.3 N/mm; 95% CI 124.9–435.6) (Table 5).

## 4. Discussion

In the current study, heavy resistance training was well tolerated by the participants, and no major adverse events were reported during the intervention period. Furthermore, a substantial reduction in knee pain was observed in all participants at follow-up. Also, the participants had improvements in knee-related quality of life and a reduction in fear of movement on a group level. These findings were accompanied by improvements in neuromuscular factors related to active knee joint stability such as lower extremity strength, quadriceps RTD, and knee proprioception, as well as increased patellar tendon stiffness.

In clinical practice, individuals with hypermobility spectrum disorders and knee pain are typically offered different combinations of low-intensity resistance training and neuromuscular exercises [17, 18]. In fact, heavy resistance training has traditionally been considered inappropriate in this population, probably due to participants' and/or health professionals' fear of pain increase, risk of injuries, or other adverse events [17, 18]. Contrary to these beliefs, we observed no major adverse events during the intervention period, and none of the four participants lost to follow-up reported any complications related to the intervention. Among the remaining 16 participants, they all completed at least 88% of the planned training sessions, demonstrating excellent adherence.

In the current study, all exercises were supervised, guided, and encouraged by trained final-year physiotherapy students or the first author; in a postintervention focus group interview, these aspects were consistently mentioned by the participants as being important for their adherence and confidence. The mean acute pain flares induced by each training session were low and did not change systematically over time, but four participants had clinical examinations by the project physiotherapist due to complaints of knee or hip pain. Therefore, successfully, temporary small adjustments of, e.g., range of motion in the leg extension exercise or the general intensity were required to reduce these overload complaints, especially when progressing from the hypertrophy phase to the heavy lifting phase (week 6). Adjustments were done on a case-by-case approach, where the intensity was reduced to a level where the pain was acceptable, i.e., lower than or equal to five on the NRS. The requested RM load was returned as soon as possible, often in the following session. Also, one participant had to perform leg curls in a supine lying position due to pain during sitting leg curls (as performed by the other participants). These minor complaints were expected, as the familiarization and hypertrophy periods were relatively short. Future studies should consider extending both phases.

Heavy resistance training improved lower extremity muscle strength in this group of young women with GHJk and knee pain. The mean improvements in knee extensor MVC of 11% are comparable to what is reported in untrained middle-aged women without GJH and without knee pain following a similar intervention [41] and in line with the observation that individuals with hypermobility spectrum disorders increase muscle strength at the same rate as controls [6]. In the current study, 13 out of 16 participants improved their RTD, which has been suggested to be an important protective compensatory factor for counteracting symptomatic joint hypermobility [42]. The observed increased lower extremity muscle strength ostensibly seems to translate into clinically important changes, since knee pain (VAS_NA_) was reduced in all 16 participants, on average by more than 30 mm at follow-up. Usually, changes in VAS pain of 15–20 mm are considered clinically relevant [40]. The current decrease in VAS_NA_ was supported by improvements in the patient-reported outcomes of KOOS and TSK, although these improvements generally were of lower magnitude and not as uniform.

Impaired knee proprioception is a consistent finding among individuals with hypermobility spectrum disorders [12–14, 19]. We found that knee proprioception, assessed as AAE, improved in all participants, on average by 55% after the intervention period, reaching values similar to populations without hypermobility [12]. Furthermore, patellar tendon stiffness increased in 11 out of 16 participants or by 11% on average, which is comparable to changes reported in healthy adult populations after comparable training interventions [22, 23]. Increased patellar tendon stiffness may be important in improving active knee joint stability by increasing control of limb positioning, joint position sense, and force transmission efficacy [43, 44]. The current improvements in lower extremity strength (5RM, MVC, RTD, SLHD), knee proprioception, and increased patellar tendon stiffness are most likely contributing to an increased active knee joint stability, besides the improvements in knee pain during a self-nominated activity. The improvements seen in KOOS and TSK on a group-level support the overall hypothesis of heavy strength training as a beneficial method for improving active knee joint stability. In the current study, the young women with knee joint hypermobility ended up being able to lift and control very heavy loads corresponding to 5RM, which for some of the participants translated to, e.g., more than 200 kg in the leg press exercise. This points to notice of the importance of applying sufficient (heavy) load to effectively develop and maximize the neuromuscular response in rehabilitation in individuals with knee joint hypermobility.

### 4.1. Limitations

As case series precludes definitive conclusions on the actual effect of the intervention, future randomized controlled trials are required. It is not known if the intervention is feasible in terms of costs or other settings with less supervision for example.

Also, as all participants included in the analyses were women, who volunteered and were highly motivated for heavy strength training, it remains unknown whether males or individuals less motivated by the current training method will experience the same magnitude of improvement.

## 5. Conclusion

In contrast to current practice and beliefs, an intervention including 12 weeks of supervised, progressive heavy resistance training was well tolerated and potentially beneficial in young women with generalised joint hypermobility, knee joint hypermobility, and knee pain. In future studies, supervised, heavy resistance training should be compared with current practice to determine the most effective and efficient intervention for this population.

## Figures and Tables

**Table 1:** Individual and mean baseline participant characteristics.

Participant ID (*n* = 16, all women)	1	2	3	4	5	6	7	8	9	10	11	12	13	14	15	16	Mean (SD)
Age (years)	22	25	21	23	21	24	24	27	23	23	25	30	24	28	22	25	24.2 (2.5)
Height (cm)	162.2	174.5	172.5	167.5	177	167	167	180	162.5	179	177	165.5	168.5	178	171.5	171.5	171.3 (5.9)
BMI (kg/m^2^)	23.7	21	34.6	21	28.1	26.8	33.9	25.6	22.9	25.3	23.8	37.9	26.1	27.3	27.2	21.1	26.6 (5.0)
Beighton test score (points 0–9)	5	6	9	5	6	6	6	5	7	9	6	6	5	7	7	7	6.4 (1.3)
Hyperextension right knee (degrees)	13	11	11	11	12	11	11	9	11	11	11	8	9	11	11	11	10.8 (1.2)
Hyperextension left knee (degrees)	16	11	11	7	11	11	11	11	12	12	11	11	11	12	12	12	11.4 (1.7)
Most aggravating activity for knee pain	R&W	PS	OA	OA	OA	PS	SC	OA	R&W	PS	PS	SC	R&W	PS	SC	SC	—

R&W = running and walking, PS = prolonged standing, SC = stair climbing, O = other activities.

**Table 2:** Individual data on participant reported outcomes.

Participant ID (*n* = 16, all women)	1	2	3	4	5	6	7	8	9	10	11	12	13	14	15	16
VAS_NA_ baseline (mm, 0–100)	57	64	79	72	68	65	65	45	41	65	35	77	70	55	80	60
VAS_NA_follow-up (mm, 0–100)	30	55	35	60	10	45	10	20	28	64	10	12	39	30	10	20
VAS_NA_ change (%)	47.4	14.1	55.7	16.7	85.3	30.8	84.6	55.6	31.7	1.5	71.4	84.4	44.3	45.5	87.5	66.7
VAS_LW_ baseline (mm, 0–100)	40	16	48	8	37	15	10	45	64	30	10	62	50	30	18	25
VAS_LW_follow-up (mm, 0–100)	20	25	30	0	4	10	10	20	28	24	10	25	26	30	15	20
VAS_LW_ change (%)	50	−56	38	100	89	33	0	56	56	20	0	60	48	0	17	20
KOOS symptoms baseline (points, 0–100)	96.4	92.9	92.9	67.9	85.7	71.4	100	62.9	67.9	85.7	100	46.4	75	71.4	75	82.1
KOOS symptoms follow-up (points, 0–100)	96.4	89.3	82.1	78.6	89.3	82.1	92.9	69.4	57.1	71.4	100	82.1	85.7	89.3	67.9	89.3
KOOS symptoms change (%)	0.0	−3.9	−11.6	15.8	4.2	15.0	−7.1	10.3	−15.9	−16.7	0.0	76.9	14.3	25.1	−9.5	8.8
KOOS pain baseline (points, 0–100)	72.2	75	58.3	77.8	72.2	77.8	94.4	86.1	69.4	63.9	80.6	61.1	55.6	88.9	55.6	77.8
KOOS pain follow-up (points, 0–100)	88.9	75	72.2	86.1	88.9	91.7	88.9	82.1	77.8	66.7	86.1	83.3	75	75	80.6	77.8
KOOS pain change (%)	23.1	0.0	23.8	10.7	23.1	17.9	−5.8	−4.6	12.1	4.4	6.8	36.3	34.9	−15.6	45.0	0.0
KOOS adl baseline (points, 0–100)	92.7	94.1	86.8	89.7	82.4	82.4	98.5	92.7	88.2	82.4	94.1	69.1	80.9	80.9	80.9	92.6
KOOS adl follow-up (points, 0–100)	97.1	86.8	83.8	95.6	98.5	91.2	98.5	86.8	89.7	83.8	97.1	92.6	94.1	89.7	82.4	89.7
KOOS adl change (%)	4.7	−7.8	−3.5	6.6	19.5	10.7	0.0	−6.4	1.7	1.7	3.2	34.0	16.3	10.9	1.9	−3.1
KOOS sport baseline (points, 0–100)	65	90	55	30	50	50	100	55	45	85	50	60	45	55	25	75
KOOS sport follow-up (points, 0–100)	65	70	45	40	83.3	80	95	50	45	85	93.8	66.7	70	100	45	75
KOOS sport change (%)	0.0	−22.2	−18.2	33.3	66.6	60.0	−5.0	−9.1	0.0	0.0	87.6	11.2	55.6	81.8	80.0	0.0
KOOS qol baseline (points, 0–100)	62.5	68.8	50	25	37.5	50	62.5	56.3	56.3	43.8	43.8	31.3	43.8	56.3	43.8	75
KOOS qol follow-up (points, 0–100)	75	56.3	50	50	50	75	75	50	56.3	43.8	75	75	56.3	56.3	43.8	81.3
KOOS qol change (%)	20.0	−18.2	0.0	100.0	33.3	50.0	20.0	−11.2	0.0	0.0	71.2	139.6	28.5	0.0	0.0	8.4
TSK baseline (points, 17–65)	43	30	43	31	43	31	39	31	41	36	39	43	42	42	40	39
TSK follow-up (points, 17–65)	33	34	42	29	34	30	32	31	40	35	32	29	35	34	35	39
TSK change (%)	23.3	−13.3	2.3	6.5	20.9	3.2	17.9	0.0	2.4	2.8	17.9	32.6	16.7	19.0	12.5	0.0

VAS_NA_ = the participants pre-nominated activity, being the most aggravating for their knee joint pain. VAS_LW_ = average knee pain during the last week. KOOS = Knee injury and osteoarthritis outcome score, five subscales: symptoms, pain, function in daily living (adl), function in sport and recreation (sport) and knee related quality of life (qol). TSK = tampa scale of kinesiophobia, self-reported fear of movement.

**Table 3:** Mean participant reported outcome measures.

Outcome measure	Baseline mean (SD)	Follow-up mean (SD)	Difference (95% CI)	*P* value
VAS_NA_ (mm, 0–100)	62.4 (13.2)	29.9 (18.5)	32.5 (21.4–43.6)	<0.001
VAS_LW_ (mm, 0–100)	31.8 (18.4)	18.6 (9.4)	13.2 (5.5–20.9)	0.002
KOOS pain (points, 0–100)	72.9 (11.8)	81.0 (7.2)	8.0 (2.2–13.9)	0.010
KOOS symptoms (points, 0–100)	79.6 (15.0)	82.7 (11.5)	3.08 (−3.7–9.9)	0.35
KOOS ADL (points, 0–100)	86.8 (7.5)	91.1 (5.4)	4.3 (−0.1–8.7)	0.050
KOOS sport/Rec (points, 0–100)	58.4 (20.5)	69.3 (19.7)	10.9 (0.4–21.3)	0.043
KOOS QOL (points, 0–100)	50.4 (13.4)	60.6 (13.0)	10.2 (2.2–18.1)	0.016
TSK (points, 17–65)	38.3 (4.9)	34.0 (3.8)	4.3 (1.8–6.8)	0.002

VAS_NA_ = the participants pre-nominated activity, being the most aggravating for their knee joint pain. VAS_LW_ = average knee pain during the last week. KOOS = knee injury and osteoarthritis outcome score, five subscales: pain, symptom, function in daily living (adl), function in sport and recreation (sport) and knee related quality of life (qol). TSK = tampa scale of kinesiophobia, self-reported fear of movement.

**Table 4:** Individual data on muscle strength, patellar tendon stiffness and proprioception.

Participant ID (*n* = 16, all women)	1	2	3	4	5	6	7	8	9	10	11	12	13	14	15	16
Quadridceps MVC baseline (Nm)	187.3	209.5	188.6	163.4	200.2	176.0	172.4	177.8	146.2	176.0	153.3	157.7	110.6	168.0	197.8	170.7
Quadriceps MVC follow-up (Nm)	227.5	220.7	216.4	167.3	244.0	204.2	189.0	188.0	157.1	186.8	174.7	168.6	114.8	194.9	211.1	178.8
Quadriceps MVC change (%)	21.5	5.3	14.7	2.4	21.9	16.0	9.6	5.7	7.4	6.1	13.9	6.9	3.9	16.1	6.7	4.8
RTD 0–200 ms baseline (Nm/s)	666.6	892.5	712.3	706.0	452.1	738.0	542.5	744.5	648.8	714.8	637.4	733.2	468.1	764.5	682.6	685.8
RTD 0–200 ms follow-up (Nm/s)	978.9	782.6	876.9	584.3	634.3	838.6	824.6	800.6	660.8	727.8	729.7	737.8	474.2	740.0	905.0	705.3
RTD 0–200 ms change (%)	46.8	−12.3	23.1	−17.2	40.3	13.6	52.0	7.5	1.9	1.8	14.5	0.6	1.3	−3.2	32.6	2.9
5RM leg press baseline (kg)	127.5	145	80	75	120	95	120	105	110	107.5	80	95	90	90	150	100
5RM leg press follow-up (kg)	230	210	245	145	160	150	190	140	130	150	135	145	110	145	190	150
5RM leg press change (%)	80.4	44.8	206.3	93.3	33.3	57.9	58.3	33.3	18.2	39.5	68.8	52.6	22.2	61.1	26.7	50.0
5RM leg curl baseline (kg)	37.5	42.5	35	30	50	40	35	52.5	35	45	35	30	27.5	40	40	30
5RM leg curl follow-up (kg)	45	60	50	45	62.5	52.5	60	62.5	45	70	50	55	37.5	50	57.5	40
5RM leg curl change (%)	20.0	41.2	42.9	50.0	25.0	31.3	71.4	19.0	28.6	55.6	42.9	83.3	36.4	25.0	43.8	33.3
5RM leg extension baseline (kg)	40	35	45	40	62.5	55	75	65	42.5	40	50	45	50	70	52.5	50
5RM leg extension follow-up (kg)	105	102.5	80	85	102.5	85	100	109	70	80	90	80	62.5	—	121.5	85
5RM leg extension change (%)	162.5	192.9	77.8	112.5	64.0	54.5	33.3	67.7	64.7	100.0	80.0	77.8	25.0	—	131.4	70.0
5RM sitting calf raises baseline (kg)	160	135	110	100	125	160	135	145	135	185	110	90	120	150	150	145
5RM sitting calf raises follow-up (kg)	260	200	220	200	250	185	200	230	190	263	160	210	195	195	220	170
5RM sitting calf raises change (%)	62.5	48.1	100.0	100.0	100.0	15.6	48.1	58.6	40.7	42.2	45.5	133.3	62.5	30.0	46.7	17.2
Single leg hop for distance baseline (cm)	148	150	84	116	79	112	85	118	104	108	77	40	72	113	131	120
Single leg hop for distance follow-up (cm)	169	154	80	128	92	127	96	155	114	129	105	56	96	132	156	136
Single leg hop for distance change (%)	14.2	2.7	−4.8	10.3	16.5	13.4	12.9	31.4	9.6	19.4	36.4	40.0	33.3	16.8	19.1	13.3
Patellar tendon stiffness baseline (N/mm)	3344	2343	1861	2700	2143	2095	3705	2919	2052	1603	3040	3239	2324	2601	2517	2257
Patellar tendon stiffness follow-up (N/mm)	3847	2517	2326	2671	2610	2042	3557	3342	2936	1921	3342	3476	2832	2588	2407	2813
Patellar tendon stiffness change (%)	15.0	7.4	25.0	−1.1	21.8	−2.5	−4.0	14.5	43.1	19.8	9.9	7.3	21.9	−0.5	−4.4	24.6
Proprioception, AAE baseline (degrees)	5.0	2.3	3.5	4.8	3.4	2.8	4.0	3.0	5.8	5.5	2.5	5.5	6.0	3.5	3.3	3.2
Proprioception, AAE follow-up (degrees)	1.5	1.2	1.7	1.8	1.2	2.3	1.3	2.2	1.7	1.5	0.8	3.2	2.3	1.8	3.1	1.0
Proprioception, AAE change (%)	70.0	49.8	52.3	62.5	65.4	17.7	67.5	26.7	70.8	72.7	66.8	41.8	61.7	47.7	6.9	68.5

RM = repetition maximum, MVC = maximal voluntary contraction, RTD = rate of torque development, SLDH = single hop for distance, AAE = mean absolute angle error in a knee joint position reproduction test.

**Table 5:** Mean data on muscle strength, patellar tendon stiffness and proprioception.

	Baseline mean (SD)	Follow-up mean (SD)	Absolute difference (95% CI)	Difference (%)	*P* value
5RM leg press (kg)	105.6 (22.3)	164.1 (37.9)	58.4 (39.9–77.0)	55.3	<0.001
5RM leg curl (kg)	37.8 (7.2)	52.7 (9.0)	14.8 (11.7–17.9)	39.1	<0.001
5RM leg extension (kg)	49.8 (10.9)	90.5 (15.9)	40.7 (31.6–49.5)	81.7	<0.001
5RM sitting calf raises (kg)	134.7 (24.8)	209.3 (29.9)	74.6 (58.1–91.0)	55.4	<0.001
Knee extensor MVC (Nm)	172.2 (23.9)	190.2 (31.3)	18.0 (11.6–24.5)	10.5	<0.001
RTD 0–200 ms (Nm/s)	674.4 (110.7)	750.1 (125.8)	75.7 (7.6–143.8)	11.2	0.030
SLHD (cm)	103.6 (29.2)	120.3 (31.2)	16.8 (11.6–21.9)	16.2	<0.001
Proprioception, AAE (degrees)	4.0 (1.2)	1.8 (0.7)	2.2 (1.6–2.9)	55.0	<0.001
Patellar tendon stiffness (N/mm)	2546.4 (578.1)	2826.6 (555.3)	280.3 (124.9–435.6)	11.0	0.002

RM = repetition maximum, MVC = maximal voluntary contraction, RTD = rate of torque development, SLDH = single hop for distance, AAE = mean absolute angle error in a knee joint position reproduction test.

## Data Availability

The data used to support this study are available from the corresponding author upon request.

## Supplementary Materials

Supplementary Materials The intervention is reported according to the TIDieR guidelines. The heavy strength training program is described according to the CERT guidelines. Appendix A: training programme. Appendix B: technical details.
