# Supplementary material for: Supervised, Heavy Resistance Training Is Tolerated and Potentially Beneficial in Women with Knee Pain and Knee Joint Hypermobility: A Case Series
Source: Transl Sports Med. 2022 Dec 30;2022:8367134. doi: 10.1155/2022/8367134 (PMC11022762; doi:10.1155/2022/8367134)
Supplement: Supplementary Materials — The intervention is reported according to the TIDieR guidelines. The heavy strength training program is described according to the CERT guidelines. Appendix A: training programme. Appendix B: technical details. [file 8367134.f1.zip › Appendix A_training programme.docx]

**Appendix A**

*Training programme*

*Overview of the supervised, heavy progressive resistance training programme performed twice a week. All sessions lasted between 60 and 90 minutes and took place at the university training centre. RM=Repetition Maximum.*

|  | Week 1 | Week 2 | Week 3-5 | Week 6-8 | Week 9-11 | Week 12 |
| --- | --- | --- | --- | --- | --- | --- |
| Aim | Familiarisation | Familiarisation | Hypertrophy | Strength I | Strength II | Tapering |
| Sets | 3 | 3 | 3 | 4 | 4 | 3 |
|  |  | | |  | | |
| Intensity  (intended) | 14 RM | 12 RM | 10 RM | 9-8-7-6 RM | 8-7-6-5 RM | 10 RM |
| Repetitions  (intended) | 10 | 10 | 10 | 9-8-7-6 | 8-7-6-5 | 6 |
| Pause  between sets | 1-1.5 min. | 1-1.5 min. | 1-1.5 min. | 2-3 min. | 2-3 min. | 2-3 min. |
| Focus | Correct technique  No hyperextension of the knee | Correct technique  No hyperextension of the knee | Every set to  failure  Encouraged by the supervisor | Focus on explosive concentric contraction and controlled eccentric phase  Every set to failure | Focus on explosive concentric contraction and controlled eccentric phase  Every set to failure | Feeling of being in excess |
| Exercises | Leg press, sitting calf raises, leg extension, leg curl and forward lunges (or other easier lunge variations, depending on skills) | | | Leg press, sitting calf raises, leg extension, leg curl and forward lunges | | |
