# Supplementary material for: Supervised, Heavy Resistance Training Is Tolerated and Potentially Beneficial in Women with Knee Pain and Knee Joint Hypermobility: A Case Series
Source: Transl Sports Med. 2022 Dec 30;2022:8367134. doi: 10.1155/2022/8367134 (PMC11022762; doi:10.1155/2022/8367134)
Supplement: Supplementary Materials — The intervention is reported according to the TIDieR guidelines. The heavy strength training program is described according to the CERT guidelines. Appendix A: training programme. Appendix B: technical details. [file 8367134.f1.zip › Appendix B_technical details.docx]

**Appendix B**

*Additional technical details regarding treatment of force signals and rate of torque development measurements.*

Force data were amplified (gain 100) and filtered with a 400 Hz lowpass filter by a bridge amplifier (CED 1902) and sampled with CED micro 1401 (Cambridge Electronic Design Limited, Cambridge ENGLAND) at 2000Hz (16bit) using software Spike2 (ver.5.13).

RTD analysis was performed in MATLAB (7.5.0 (R2007b)) where data were further filtered with a 15 Hz lowpass digital filter (fourth-order Butterworth, zero-phase). To ensure that baseline and follow-up measurements were comparable, onset of muscle contraction was defined as the time point where torque reached two percent of the MVC attained in each attempt.
