# Supplementary material for: Supervised, Heavy Resistance Training Is Tolerated and Potentially Beneficial in Women with Knee Pain and Knee Joint Hypermobility: A Case Series
Source: Transl Sports Med. 2022 Dec 30;2022:8367134. doi: 10.1155/2022/8367134 (PMC11022762; doi:10.1155/2022/8367134)
Supplement: Supplementary Materials — The intervention is reported according to the TIDieR guidelines. The heavy strength training program is described according to the CERT guidelines. Appendix A: training programme. Appendix B: technical details. [file 8367134.f1.zip › Description of the Supplementary Materials.docx]

**Supplementary Materials**

The intervention is reported according to the TIDieR guidelines.

The heavy strength training program is described according to the CERT guidelines.

Appendix A_training programme.

Appendix B_technical details
